# Rôle de la radiothérapie dans le traitement de l'améloblastome: à propos de deux cas

**DOI:** 10.11604/pamj.2014.19.89.4491

**Published:** 2014-09-26

**Authors:** Abderrahman El Mazghi, Touria Bouhafa, Hanan El Kacemi, Kaoutar Loukili, Laila Chbani, Taieb Kebdani, Khalid Hassouni

**Affiliations:** 1 Service de Radiothérapie, CHU Hassan II, Fès, Maroc; 2 Service de Radiothérapie, Institut national d'Oncologie, Rabat, Maroc; 3 Service d'Anatomie Pathologique, CHU Hassan II, Fès, Maroc

**Keywords:** Améloblastome, mandibule, radiothérapie, tumeur bénigne, Ameloblastoma, mandible, radiotherapy, benign tumor

## Abstract

L'améloblastome est une tumeur odontogène bénigne mais à pouvoir agressif et invasif local important. C'est une tumeur rare, elle représente 1% des tumeurs des maxillaires. Le rôle de la radiothérapie dans son traitement est actuellement démontré pour les tumeurs inopérables. Nous rapportons 2 cas d'améloblastomes mandibulaires chez deux patients qui ont bénéficié d'une radiothérapie externe à la dose de 60 Gy. L’évolution a été marquée par une rémission complète de la maladie dans les deux cas avec un recul de 2 et 5 ans.

## Introduction

L'améloblastome est une tumeur bénigne odontogénique rare, localement agressive qui se localise habituellement dans le voisinage des molaires ou des branches mandibulaires. Elle représente 1% des tumeurs des maxillaires. Son traitement est pluridisciplinaire. L’évolution est marquée par la fréquence des récidives locales. Incontrôlé, l'ameloblastome peut entraîner une morbidité importante et parfois la mort, toutefois, correctement traité, son pronostic reste bon [1, 2]. A travers ces deux nouveaux cas traités par radiothérapie externe, nous allons discuter des aspects diagnostiques et thérapeutiques de cette tumeur rare en se concentrant sur le rôle et l'efficacité de la radiothérapie dans sa prise en charge.

## Patient et observation

### Cas 1

**Figure 1:**
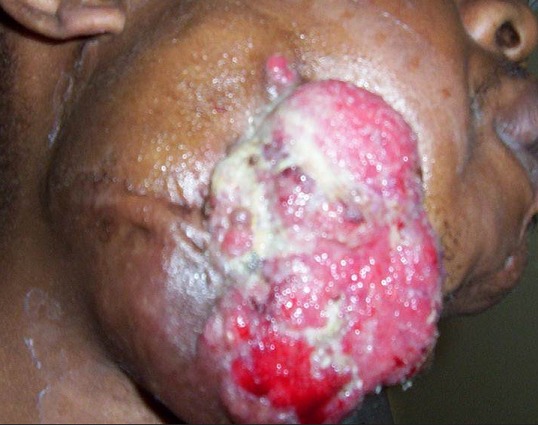
Photo de profil montrant une volumineuse tumeur ulcero-bourgeonnante cervico-jugale droit de 18x12cm avec effraction cutanée et saignement spontané

**Figure 2:**
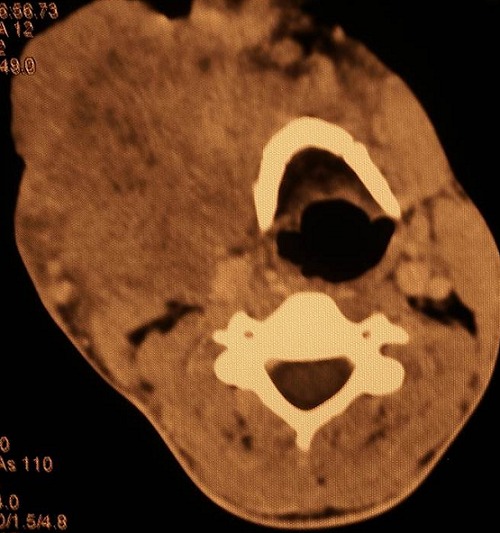
Coupe axiale de la TDM cervico-faciale montrant un volumineux processus lésionnel nécrosé et ulcéré de la région latéro-cervicale droite

Il s'agit d'un patient de 25 ans, tabagique chronique, qui a bénéficié d'une hémi-mandibulectomie droite pour améloblastome mandibulaire évoluant depuis plusieurs années. 3 ans plus tard, il s'est présenté au service avec une récidive locale confirmée histologiquement et négligée par le patient. A l'examen clinique il s'agissait d'une volumineuse tumeur ulcero-bourgeonante cervico-jugale droite de 18x12cm avec effraction cutanée et saignement spontané (Figure 1) sans adénopathies palpables. Une TDM cervico-faciale a montré un volumineux processus lésionnel nécrosé et ulcéré de la région latéro-cervicale droite, infiltrant la peau et la graisse sous cutanée, envahissant l'hémilangue et bourgeonnant dans la lumière pharyngo-laryngée (Figure 2). La TDM thoracique était normale. Le traitement a consisté en radiothérapie externe aux photons X de haute énergie, à la dose totale 60 Gy à raison de 2 Gy /séance, 5 séances/semaine en 6 semaines avec une bonne tolérance. L’évolution était marquée par une rémission complète et l'absence de récidive après un suivi post- thérapeutique de 2 ans.

### Cas 2

**Figure 3:**
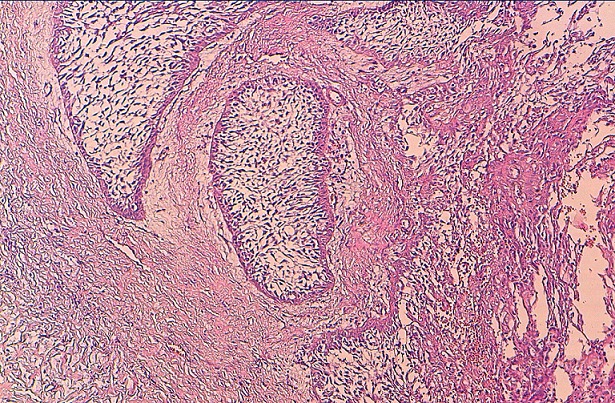
HES X250: lobules de cellules épithéliales disjonctives au centre entourées d'un stroma conjonctif vascularisé évoquant un améloblastome

Il s'agit d'un patient de 72 ans, tabagique chronique, sans antécédents pathologiques particuliers qui a présenté une ulcération gingivale augmentant progressivement de volume, ce qui a motivé une consultation au service d'oto-rhino-laryngologie (ORL) où une biopsie a conclu en un améloblastome sans signe d'invasion. Le patient a refusé le traitement chirurgical et a négligé sa maladie. 7 ans plus tard, il s'est présenté avec une énorme tumeur de la branche horizontale de la mandibule droite dont la radio panoramique dentaire a montré une volumineuse lésion ostéolytique cloisonnée déformant les contours externes de la branche montante de la mandibule. La TDM cervico-faciale parlait d'un processus occupant la branche horizontale de la mandibule droite soufflant et lysant la corticale avec infiltration des parties molles péri-osseuses, faisant 11 cm de grand axe, et sans adénopathies satellites. La re-biopsie avec étude anatomopathologie parle du même aspect histologique que la première biopsie (Figure 3). La radiographie pulmonaire était normale. Après discussion en réunion de concertation pluridisciplinaire des tumeurs ORL, le patient a été récusé sur le plan chirurgical et un traitement à base de radiothérapie externe 3D à la dose de 60 Gy en 6 semaines à raison de 2 Gy /séance et 5 séances/semaine par 2 champs latéraux aux photons X de haute énergie d'un accélérateur linéaire. L’évolution est marquée par une rémission complète et absence de récidive après un suivi post thérapeutique de 5 ans.

## Discussion

Le terme d'améloblastome a été suggéré par Ivy Churchill en 1930 pour remplacer le terme « adamantinome » proposé par Malassez en 1885. C'est une tumeur odontogène bénigne à caractère invasif justifiant un diagnostic précoce. L’âge médian de survenue est de 35 ans, les deux sexes sont également affectés, avec une prédominance chez la race noire pour certaines études. La majorité des améloblastomes sont polykystique et sont plus difficiles à éradiquer que les variétés mono-kystiques et périphériques [1]. Le maxillaire inférieur est atteint dans 80% des cas. La tumeur est généralement peu symptomatique et indolore, Les circonstances de découverte sont dominées par les déformations faciales ou les chutes dentaires, L'image radiologique caractéristique en: «bulles de savon» traduisant une destruction osseuse poly-géodique soufflant la corticale osseuse. Bien que la chirurgie soit la pierre angulaire du traitement, l’étendue de la résection est controversée. Les résections radicales, y compris la mandibulectomie marginale et segmentaire, entraînent des taux de contrôle local dépassant 90%. En revanche, les procédures conservatrices telles que l’énucléation et le curetage entrainent des taux de contrôle local d'environ 80% et 50% pour les améloblastomes mono-kystique et multi-kystiques respectivement [2].

La plupart des auteurs considèrent que l'améloblastome est radiorésistant alors qu’ en 1982, Reynolds a écrit un important article sur l'effet de l'irradiation sur l'améloblastome dans lequel il a discuté les principes de base de la radiothérapie et a conclu que la radiothérapie a une place dans les tumeurs localement avancées ou en cas de refus de la chirurgie [3]. Un deuxième document important est celui d'Atkinson en 1984, qui a publié une série de 10 cas d'ameloblastomes traités par radiothérapie, il conclu, sur la base de son expérience et la revue de la littérature que les améloblastomes sont radiosensibles [4]. Depuis lors, il y a peu de cas publiés dans la littérature sur le rôle de la radiothérapie comme modalité utile dans le traitement des ameloblastomas [5–7]. Rastogi a publié en 2006 un cas d'ameloblastome localement avancée de la mandibule qui a bien repondu à une radiothérapie externe à la dose de 60 Gy en 30 séances de 2 Gy au Cobalt 60 par deux champs lateraux. Ce malade est resté en bon cotrol locale deux ans après la fin du traitement [8]. Nous, ici présentons deux cas d'ameloblastomes localement avancés et supportons l'idée que l'améloblastome est une tumeur intrinsèquement radiosensible surtout au rayonnement de haute énergie. Par conséquent, la radiothérapie moderne peut jouer un rôle important dans le traitement de ces tumeurs, en particulier dans les cas où une exérèse chirurgicale complète serait techniquement difficile ou de contre indication médicale de la chirurgie. La radiothérapie peut également augmenter la probabilité de contrôle local chez les quelques patients opérés avec des limites tumorales ou marginales.

## Conclusion

L'améloblastome est une tumeur odontogène bénigne, agressive et récidivante qui nécessite un diagnostic précoce et un traitement adéquat. La radiothérapie joue un rôle important dans la prise en charge des stades localement avancés et inopérables.
